# A novel DSN‐based fluorescence assay for MicroRNA‐133a detection and its application for LVH diagnosis in maintenance hemodialysis patients

**DOI:** 10.1002/jcla.23438

**Published:** 2020-07-16

**Authors:** Pei Huang, Xuedan Guo, Yan Jin, Qing Huang

**Affiliations:** ^1^ Department of Oncology Wuxi No. 2 People’s Hospital Affiliated to Nanjing Medical University Wuxi China; ^2^ Department of Gastroenterology Wuxi No. 2 People’s Hospital Affiliated to Nanjing Medical University Wuxi China; ^3^ Department of Emergency Wuxi People’s Hospital Affiliated to Nanjing Medical University Wuxi China

**Keywords:** DSN‐based fluorescence assay, left ventricular hypertrophy, maintenance hemodialysis, MicroRNA‐133a

## Abstract

Left ventricular hypertrophy (LVH) is the most powerful predictor of cardiovascular mortality in maintenance hemodialysis (MHD) patients. Circulating microRNA‐133a (miR‐133a) was reported to be a potential biomarker for LVH in MHD patients. The aim of this experiment is to establish a novel DSN (duplex‐specific‐nuclease)‐based fluorescence assay for the ultrasensitive detection of miR‐133a and investigate its application for LVH diagnosis in MHD patients. The results indicate DSN enzyme combined with ultrathin metallic MoS_2_ nanosheets presents high sensitivity, specificity, and low fluorescence background for miR‐133a detection. Then, circulating miR‐133a levels in plasma from 40 MHD patients and 20 healthy controls are analyzed by such assay. The levels of miR‐133a are down‐regulated in MHD patients with LVH compared to MHD patients without LVH and healthy controls, and the ROC (receiver operating characteristic) curve shows strong separation between MHD with LVH patients and MHD without LVH patients. Furthermore, the liner regression analysis shows negative correlation of miR‐133a level and interventricular septum thickness (IVS) as well as left ventricular mass index (LVMI), the indicators of LVH. Therefore, our findings reveal DSN‐based fluorescence assay for miR‐133a is suitable for LVH diagnosis in MHD patients.

## Graphical abstract

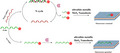

## 1 INTRODUCTION

Cardiovascular disease (CVD), with a high morbidity and mortality, is the leading cause of death for maintenance hemodialysis (MHD) patients with end‐stage renal disease (ESRD).^1^, ^2^, ^3^ The major frequent causes for cardiac death are cardiomyopathy and ischemic heart disease.^4^ Left ventricular hypertrophy (LVH) contributes to most cardiovascular mortalities of MHD patients and is an independent risk factor for survival in this population.^5^, ^6^ Therefore, LVH is an important predictive and prognostic marker of MHD because of its established clinical value. Electrocardiographic (ECG) method for the detection of LVH is used in the clinical setting; however, the use of ECG is somewhat limited by its poor sensitivity in dialysis patients.^7^, ^8^, ^9^ Echocardiography represents a valuable method for the detection of LVH due to its wide availability and its relatively low cost; however, the main limitation is represented by the low spatial resolution and reproducibility.^10^ Although new markers of cardiac risk such as cardiac hormone brain natriuretic peptide (BNP), troponin T (cTnT), and troponin I (cTnI) draw extensive attention, the detection of these biomarkers is still scarcely applied for clinical practice in dialysis patients.^11^

MicroRNAs (miRNAs) are endogenous small non‐coding RNAs (18‐25 nucleotides) that regulate gene expression at the post‐transcriptional level.^12^ MiRNAs have been shown to play important roles in multiple biological processes and aberrant expressions of which in tissues and cells can promote various diseases.^13^, ^14^ Thus, miRNAs in circulation have been proposed as being useful in diagnostics as biomarkers for diseases and different types of disease. Existed evidence has shown that miRNAs are key modulators of cardiovascular function, and several of them have been confirmed to be useful biomarkers for cardiovascular diseases.^15^, ^16^ MiR‐133a with high expression in cardiac and skeletal muscle is dysregulated during heart hypertrophy and failure. Several studies have reported circulating miR‐133a is changed in patients with cardiac dysfunction. Study from Wen et al have revealed that miR‐133a is an effective biomarker for prediction of cardiac hypertrophy in MHD patients. The conventional used method for microRNA detection is qRT‐PCR (quantitative real‐time polymerase chain reaction); however, multiple sample processing steps and unstable characters limit its application in clinical. DSN (duplex‐specific‐nuclease)‐based fluorescence assay has emerged as a versatile component in bioanalytical strategy for miRNA detection owing to high sensitivity, specificity, short assay time, and low contamination risk. DSN shows a strong preference for digesting DNA strands in double‐stranded DNA or in DNA‐RNA hybrid duplexes. Additionally, it discriminates between fully matched and slightly mismatched short duplexes.^17^ In the present study, a novel DSN‐based fluorescence assay is established for miR‐133a detection and its application for LVH diagnosis in MHD patients is further evaluated.

## 2 MATERIALS AND METHODS

### 2.1 Clinical sample collection

This study is approved by the ethics committee of Wuxi People's Hospital and Wuxi No.2 People's Hospital. The informed consent is signed by every patient before the study. A total of 60 subjects are studied, including 40 patients with ESRD undergoing maintenance hemodialysis and 20 healthy controls. Among 40 MHD patients, 20 are diagnosed with LVH and 20 are without such complication based on ECG and echocardiography measurements. The clinical characteristics of the study population are summarized in Table 1. Blood samples are immediately collected using EDTA (ethylene diamine tetraacetic acid) tubes. All blood samples are centrifuged at 3500 g for 10 minutes within 4 hours of collection for the isolation of plasma. Then, plasma is transferred to RNase‐free tubes and stored at −80℃.

**Table 1:** Clinical characteristics of the study population

Characteristics	Healthy controls (n = 20)	MHD without LVH (n = 20)	MHD with LVH (n = 20)
Age (years)	45.25 ± 18.15	58.8 ± 16.12	58.5 ± 17.9
Sex (male/female)	8/12	12/8	13/7
Dialysis duration (months)	—	44 ± 48.69	28.95 ± 35.59
SBP (mm Hg)	122.45 ± 10.56	154.55 ± 19.83	158.65 ± 15.74
DBP (mm Hg)	71.75 ± 81.51	83.3 ± 10.84	89.65 ± 13.98
Hemoglobin (mg/dL)	13.33 ± 1.59	9.41 ± 2.36	9.31 ± 2.78
Albumin (g/dL)	39.87 ± 3.06	36.23 ± 3.95	35.78 ± 4.56
CRP (mg/L)	2.83 ± 1.89	15.91 ± 16.16**, ##	18.66 ± 29.54**, ##
LVMI (g/m^2^)	85.87 ± 28.14	89.78 ± 33.09	171.56 ± 39.35**, ##

** *P *< .01 versus healthy controls

^##^ *P *< .01 versus MHD without LVH.

### 2.2 DSN‐based fluorescence assay

Two‐steps reaction steps are conducted in this assay. In the first‐step reaction, sample from plasma (10 μL) is added to reaction mixture (100 μL) containing DSN (0.2 U), RNase inhibitor (20 U), and FAM‐labeled microRNA‐specific probe (ATTTGGTTCCATTTTACCAGCT‐FAM, 200 nmol\L). The mixture is incubated under 60℃ in a thermal cycler for 30 minutes. In the second‐step reaction, the mixture reacts with ultrathin metallic MoS_2_ nanosheets (6 μg/mL, 100 μL) under room temperature for 5 minutes. The preparation of MoS_2_ nanosheets is conducted as previously reported.^18^ A fluorescence spectrometer is used to test fluorescence emission spectra (excitation/emission maxima at 492/518 nm).

### 2.3 Statistical analysis

Biostatistical analyses are conducted by the SPSS 16.0 software package (IL, USA). All experiments are repeated three times, and data are presented as means ± SD. Difference between groups is analyzed with independent two‐sample *t* test or Mann‐Whitney *U* test. A *P < *.05 is accepted as statistically significant.

## 3 RESULTS

### 3.1 Clinical analysis of the study population

A total of 60 subjects including 40 ESRD patients with MHD treatment and 20 healthy controls without kidney and cardiac diseases are studied. Clinical characteristics of the included subjects are presented in Table 1. According to the LVMI value, forty MHD patients are divided into two groups with or without LVH. There is no significant difference between two groups with common clinical characteristics except for CRP (C‐reactive protein) and LVMI.

### 3.2 Establishment of DSN‐based fluorescence assay for miR‐133a

**Figure 1:**
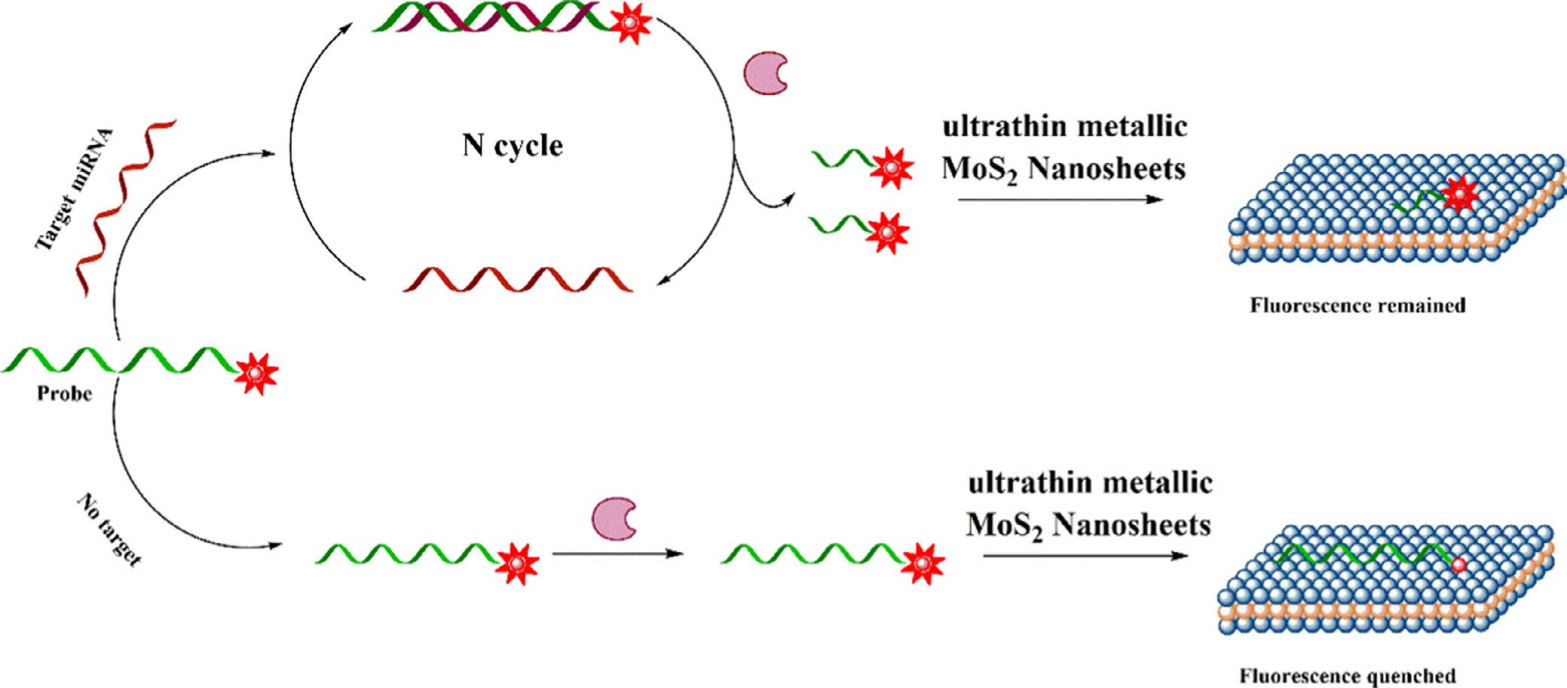
Schematic illustration of DSN‐based fluorescence assay for miR‐133a detection

**Figure 2:**
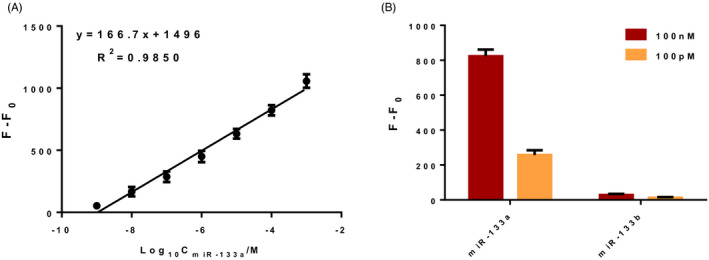
The sensitivity and specificity of DSN‐based fluorescence assay for miR‐133a. A, Sensitivity of the assay for miR‐133a detection. B, Specificity of the assay for miR‐133a detection

To determine the circulating miR‐133a levels, a novel fluorescence assay combined DSN enzyme with ultrathin metallic MoS_2_ nanosheets is designed, which is improved according to the previous study.^19^ The basis of such assay is shown as Figure 1, upon the addition of target miR‐133a, the ssDNA probe with FAM‐labeled hybridizes to the miR‐133a to form a heteroduplex of DNA/RNA. The cleavage of probe from DNA/RNA duplex occurs when DSN is added, which triggers another hybridization, cleavage and release, and leads to a large amplification. After reaction, the product incubates with ultrathin metallic MoS_2_ nanosheets and the cleaved short FAM‐labeled oligonucleotide fragments cannot be absorbed and remains a strong signal. Then, the sensitivity, linear dynamic range, and specificity are analyzed. As shown in Figure 2, the limit of detection (LOD) is approximately 1 pM according to the standard curve, and the signal from miR‐133a is stronger than that of miR‐133b, indicating high sensitivity and specificity of such assay.

### 3.3 Evaluation of DSN‐based fluorescence assay for LVH diagnosis

**Figure 3:**
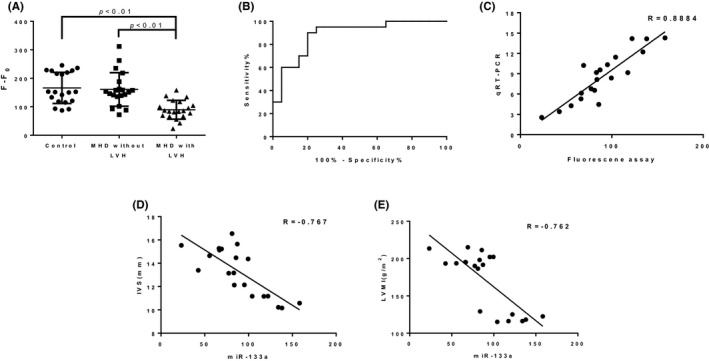
Evaluation of DSN‐based fluorescence assay for microRNA‐133a detection in LVH diagnosis. A, The comparison of miR‐133a levels between patient groups and healthy control. B, The ROC curve between two patient groups. C, The correlation analysis of miR‐133a expression from fluorescence assay and qRT‐PCR. D, The correlation between circulating miR‐133a levels and the indicators of LVH in MHD patients

MiR‐133a was previously reported to be a potential biomarker for cardiac hypertrophy in MHD patients, and in this study, the diagnostic value of miR‐133a detection based on fluorescence assay in such disease is analyzed. As shown in Figure 3A and B, the fluorescence value in MHD with LVH patients is significantly lower than the other two groups, and the ROC (receiver operating characteristic) curve shows strong separation between MHD with LVH patients and MHD without LVH patients, with an AUC (area under curve) of 0.885 (95% CI: 0.779‐0.990). In addition, comparison of DSN‐based fluorescence assay and qRT‐PCR analysis for measurement of miR‐133a level is conducted and the data show positive correlation (*R* = .8884) (Figure 3C). Then, the correlation of miR‐133a levels and the clinical factors of cardiac hypertrophy including IVS and LVMI are analyzed. As shown in Figure 3D, the liner regression analysis indicates that the fluorescence values are negatively correlated with IVS (*R* = −.767) and LVMI (*R* = −.762), the indicators of LVH.

## 4 DISCUSSION

The main renal replacement therapy for chronic uremia patients is hemodialysis; however, cardiovascular diseases, especially MHD, affect the survival rate and quality of life of this population.^20^ LVH is the most common cardiovascular complication, which is also a strong predictor of death in MHD patients.^21^ MicroRNAs are estimated to affect cellular processes by regulating expressions of key genes. Increasing evidence shows miRNAs play crucial roles during cardiovascular development and function and affect the process of cardiac hypertrophy.^22^ Extensive studies show miR‐133a presents high expression in cardiac and skeletal tissues, which is dysregulated in pathological process of cardiac growth and heart failure.^23^, ^24^ Wen et al have reported that circulating miR‐133a is considered to be the potential biomarker for cardiac hypertrophy in MHD patients.^25^ In this study, a novel DSN‐based fluorescence assay is established for miR‐133a detection and applied in the diagnosis of LVH in MHD patients.

High sensitivity efficiency makes qRT‐PCR to be the widely used method for microRNA detection, while multiple sample processing limits its application in diagnosis of heart failure.^26^ Methods based on fluorescence are recently developed for rapid detecting microRNAs; however, low sensitivity and specificity limit these methods.^27^ DSN enzyme, a nuclease isolated from hepatopancreas of the Kamchatka crab (Paralithodes camtschaticus), is widely used for signal amplification because it can cleave double‐stranded DNA or DNA in DNA/RNA duplex. Conventional semi‐conductive MoS_2_ nanosheets are combined with DSN to carry on microRNA detection because of its ability for fluorescence quenching; however, its application is currently limited due to low fluorescence quenching efficiency/fluorescence background ratio. Lan *et al* recently develop ultrathin metallic MoS_2_ nanosheets, which shows the excellent fluorescence quenching efficiency discriminative adsorption toward single‐strand DNA.^18^ Zhu et al design a novel assay for three microRNAs in acute myocardial infarction based on combination of duplex‐specific nuclease and ultrathin metallic MoS_2_ nanosheets; however, the experimental process is complicated.^19^ In this study, we improve the experimental design based on the previous study from Zhu *et al*, and our system presents high sensitivity, specificity, and low fluorescence background for miR‐133a detection, though the sensitivity and linear dynamic range is still lower and the resolution of this biosensor platform is still limited compared to qRT‐PCR. In the further study, it still needs to be improved by modifying current system.

Furthermore, a total of 60 subjects including 40 ESRD patients under MHD treatment and 20 healthy controls without kidney and cardiac diseases are enrolled and circulating miR‐133a levels are first detected by DSN‐based fluorescence assay. Our results show that the levels of circulating miR‐133a are down‐regulated in MHD with LVH patients compared to MHD patients and healthy controls, and the ROC curve shows strong separation between MHD with LVH patients and MHD without LVH patients. Furthermore, the liner regression analysis indicates the negative correlation of plasma miR‐133a levels and IVS as well as LVMI, the indictors of LVH. The significant correlation indicates DSN‐based fluorescence assay for miR‐133a is suitable for diagnosing LVH in MHD patients. In the further study, sample size needs to be expanded to verify the conclusion from this study.
